# Supplementary material for: Chirality selective magnon-phonon hybridization and magnon-induced chiral phonons in a layered zigzag antiferromagnet
Source: Nat Commun. 2023 Jun 9;14:3396. doi: 10.1038/s41467-023-39123-y (PMC10256790; doi:10.1038/s41467-023-39123-y)
Supplement: Supplementary file 1 — Supplementary Information [file 41467_2023_39123_MOESM1_ESM.pdf]

Supplementary materials for Chirality selective magnon-phonon hybridization and magnon-induced chiral phonons in a layered zigzag antiferromagnet

Jun Cui,[1,*] Emil Viñas Boström,[2,*] Mykhaylo Ozerov,[3,†] Fangliang Wu,[1] Qianni Jiang,[4] Jiun-Haw Chu,[4] Changcun Li,[5] Fucai Liu,[5] Xiaodong Xu,[4,6] Angel Rubio,[2,7,†] and Qi Zhang[1,†]

[1]*National Laboratory of Solid State Microstructures and Department of Physics, Nanjing University, Nanjing 210093, China*

[2]*Max Planck Institute for the Structure and Dynamics of Matter, Luruper Chaussee 149, 22761 Hamburg, Germany*

[3]*National High Magnetic Field Laboratory, Florida State University, Tallahassee, Florida 32310, USA*

[4]*Department of Physics, University of Washington, Seattle, Washington 98195, USA*

[5]*School of Optoelectronic Science and Engineering, University of Electronic Science and Technology of China, Chengdu, 611731 China*

[6]*Department of Materials Science and Engineering, University of Washington, Seattle, Washington 98195, USA*

[7]*Center for Computational Quantum Physics, The Flatiron Institute, New York, NY 10010, USA*

* These authors contributed equally to this work.

† Correspondence to: Q.Z. (zhangqi@nju.edu.cn), A.R. (angel.rubio@mpsd.mpg.de) and M.O. (ozerov@magnet.fsu.edu).

LIST OF CONTENT

Supplementary Figures

Figure S1: Optical anisotropy of $FePSe_3$ as a function of temperature and thicknesses.

Figure S2: Normalized magneto-infrared transmission spectra of $FePSe_3$ up to 17 T.

Figure S3: Normalized magneto-infrared transmission spectra of $FePSe_3$ up to 35 T.

Figure S4: Temperature dependent Raman spectra of $FePSe_3$ from 5 K to 200 K at zero magnetic fields.

Figure S5: Circular polarization resolved magneto-Raman spectra of $FePSe_3$.

Figure S6: Magneto-Raman spectra of magnon polarons in $FePSe_3$ at 30 K, 45 K, and 60 K.

Figure S7: Circular polarization resolved magneto-Raman spectra of AFM magnons in $FePS_3$.

Figure S8: Magnon bandstructures of $FePSe_3$.

Figure S9: Phonon displacement patterns of $FePSe_3$.

Figure S10: Magnon-induced circular phonons modes of $FePSe_3$.

Figure S11: Coherent magnon-phonon coupling in $FePSe_3$.

Figure S12: Raman circular dichroism of $FePSe_3$.

Supplementary Text

S1. Fitting of strongly coupled magnons and phonons.

S2. General Hamiltonian.

S3. Hamiltonian parameters from first principles.

S4. Magnon bandstructure and phonon modes.

S5. Magnon-phonon coupling.

S6. Magnon-polaron Hamiltonian.

S7. Magnon-induced circular phonons.

S8. Degree of circular polarization of Raman spectra.

S9. Analytical expression for the degree of circular polarization in the circular phonon limit

S10. Point group symmetries of paramagnetic and zigzag states in monolayer $FePSe_3$.

Supplementary Tables

Table S1: Magnetic parameters of $FePSe_3$.

Table S2: Phonon energies of $FePSe_3$.

Table S3: Magnetic anisotropy parameters $J_xz^{(i)}$ calculated from first principles.

Table S4: Magnetic anisotropy parameters $J_{yz}^{(i)}$ calculated from first principles.

Table S5: Phonon modulated Fe-Fe distance in $FePSe_3$.

Table S6: Magnon-Phonon coupling parameters of $FePSe_3$.

SUPPLEMENTARY FIGURES

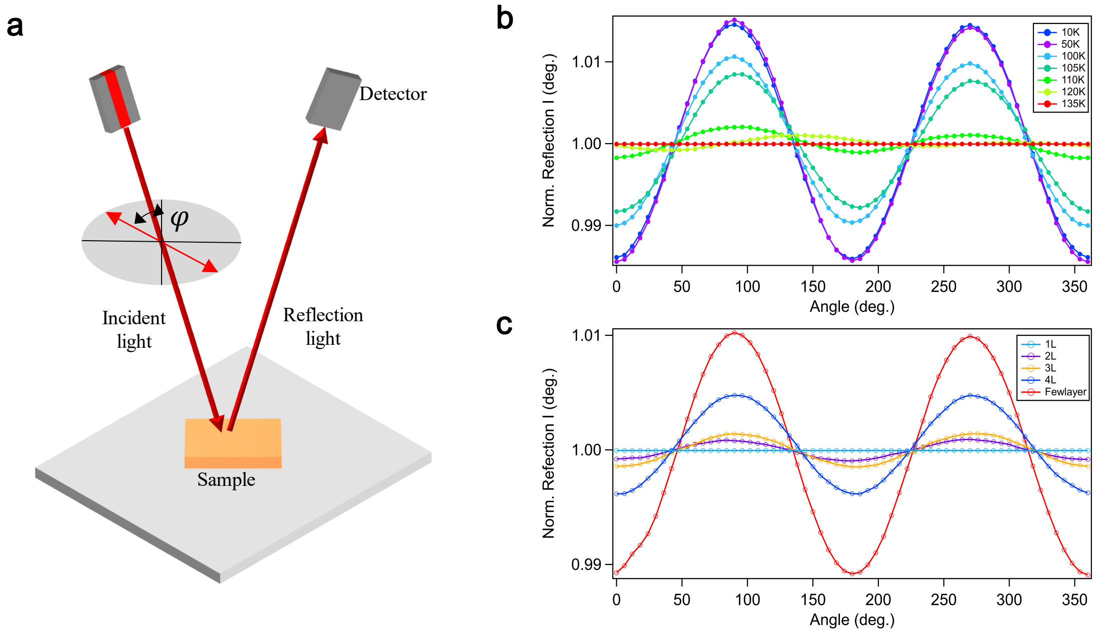

FIG. S1. **Optical anisotropy of $FePSe_3$ as a function of temperature and thicknesses.** **a**, Schematic of the linear dichroism measurement. **b**, Optical anisotropy of $FePSe_3$ at various temperatures. **c**, Optical anisotropy of $FePSe_3$ as a function of the layer number.

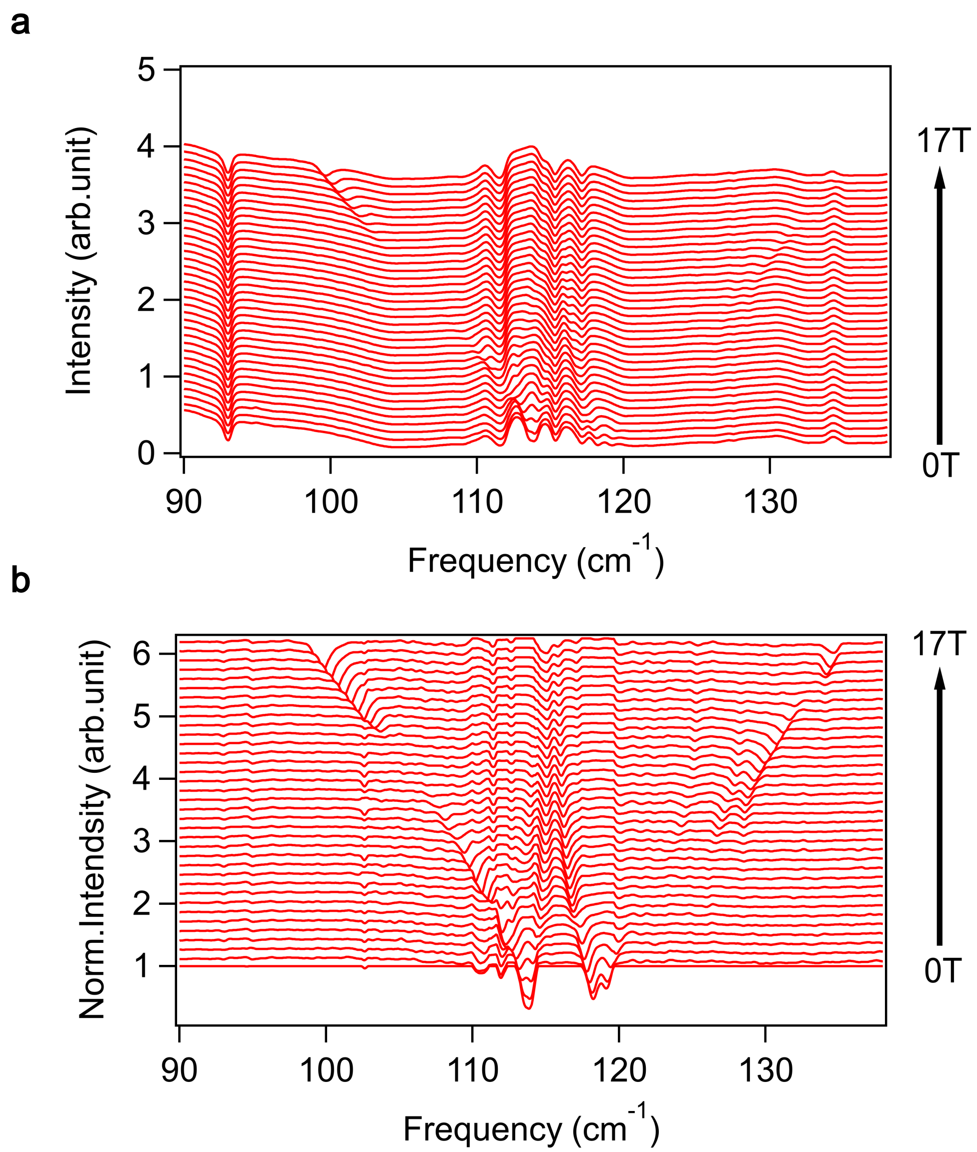

FIG. S2. **Raw and normalized magneto-infrared transmission spectra of $FePSe_3$ as a function of magnetic fields.** **a**, The raw spectra of $FePSe_3$ from 0 T to 17 T. **b** Normalized infrared transmission spectra. Raw spectra are normalized against the average of all spectra, resulting in successful suppression of field-independent background in the transmittance. All spectra are measured at 4 K and shifted for clarity.

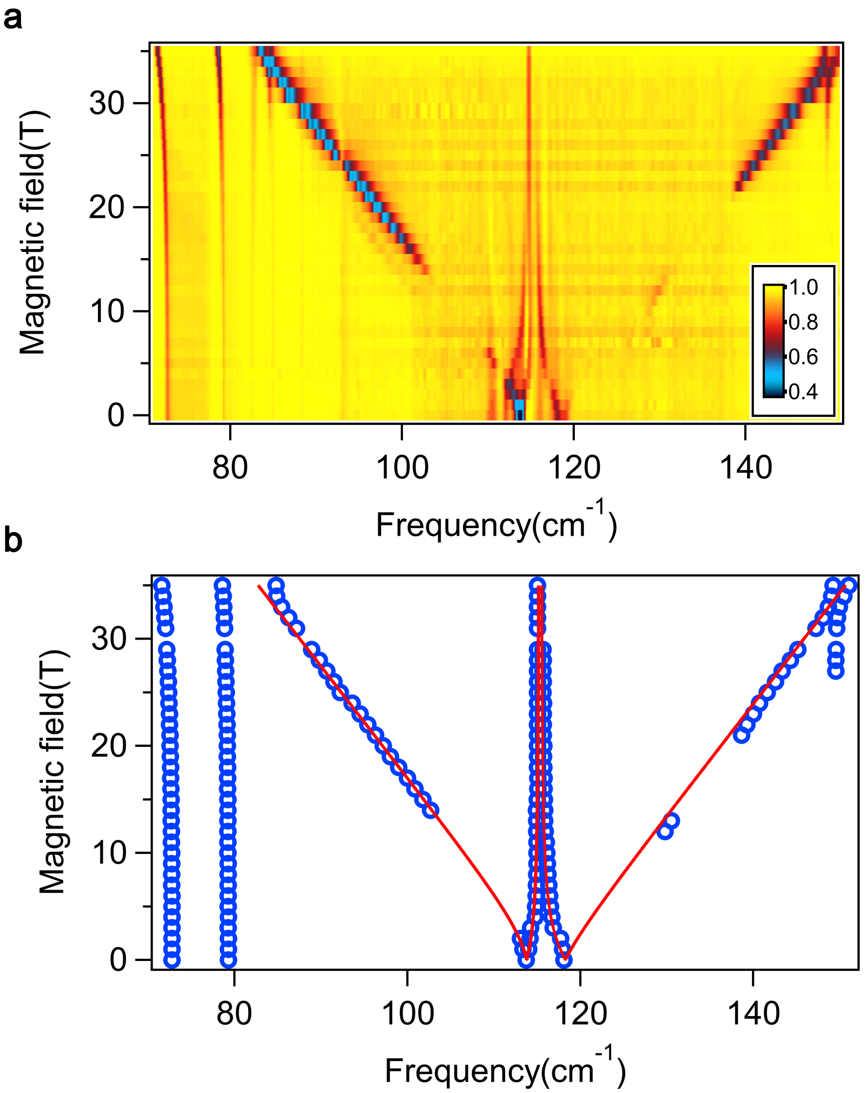

FIG. S3. **Normalized magneto-infrared transmission spectra of $FePSe_3$ up to 35 T.** **a**, Normalized magneto-infrared transmission spectra of $FePSe_3$ measured with magnetic field sweeping from 0 to 35 T. **b**, Peak position of magnon polaron branches and nearby phonon modes (blue circles). The red solid lines are the same fitting curves shown in Fig.2c. All spectra are taken at 4 K.

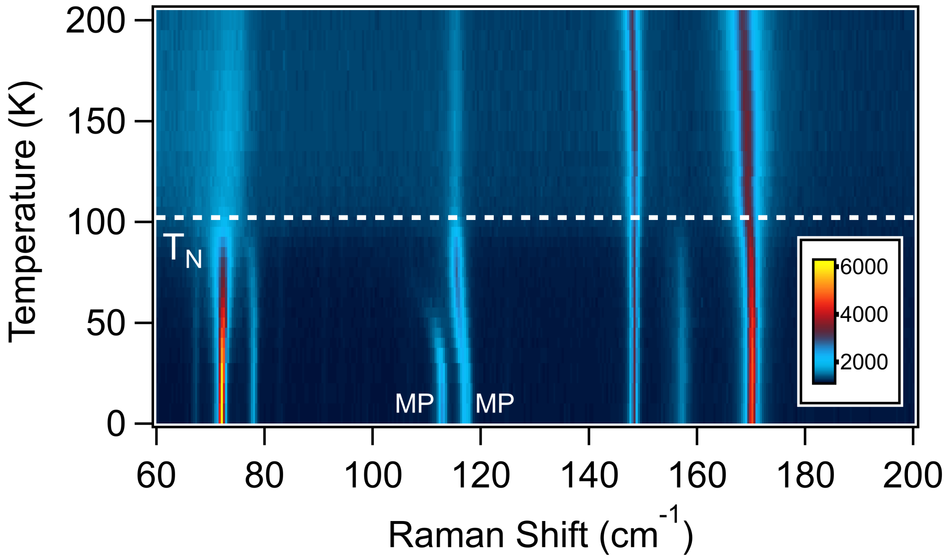

FIG. S4. **Temperature dependent Raman spectra of $FePSe_3$ from 5 K to 200 K at zero magnetic fields.** Two magnon polaron (MP) modes are marked.

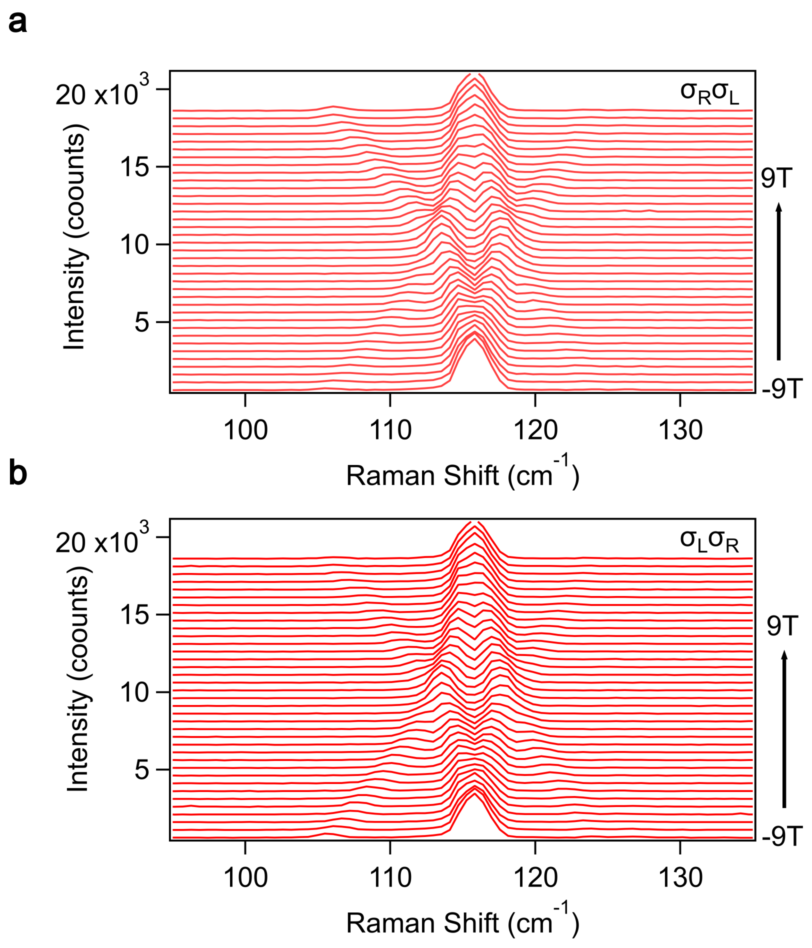

FIG. S5. **Circular polarization resolved Magneto-Raman spectra of $FePSe_3$.** cross-polarization resolved magneto-Raman spectra (from -9 T to 9 T) of $FePSe_3$. **a**, Right-handed circular excitation and left-handed detection ($\sigma_R\sigma_L$). **b**, Left-handed circular excitation and right-handed detection ($\sigma_L\sigma_R$).

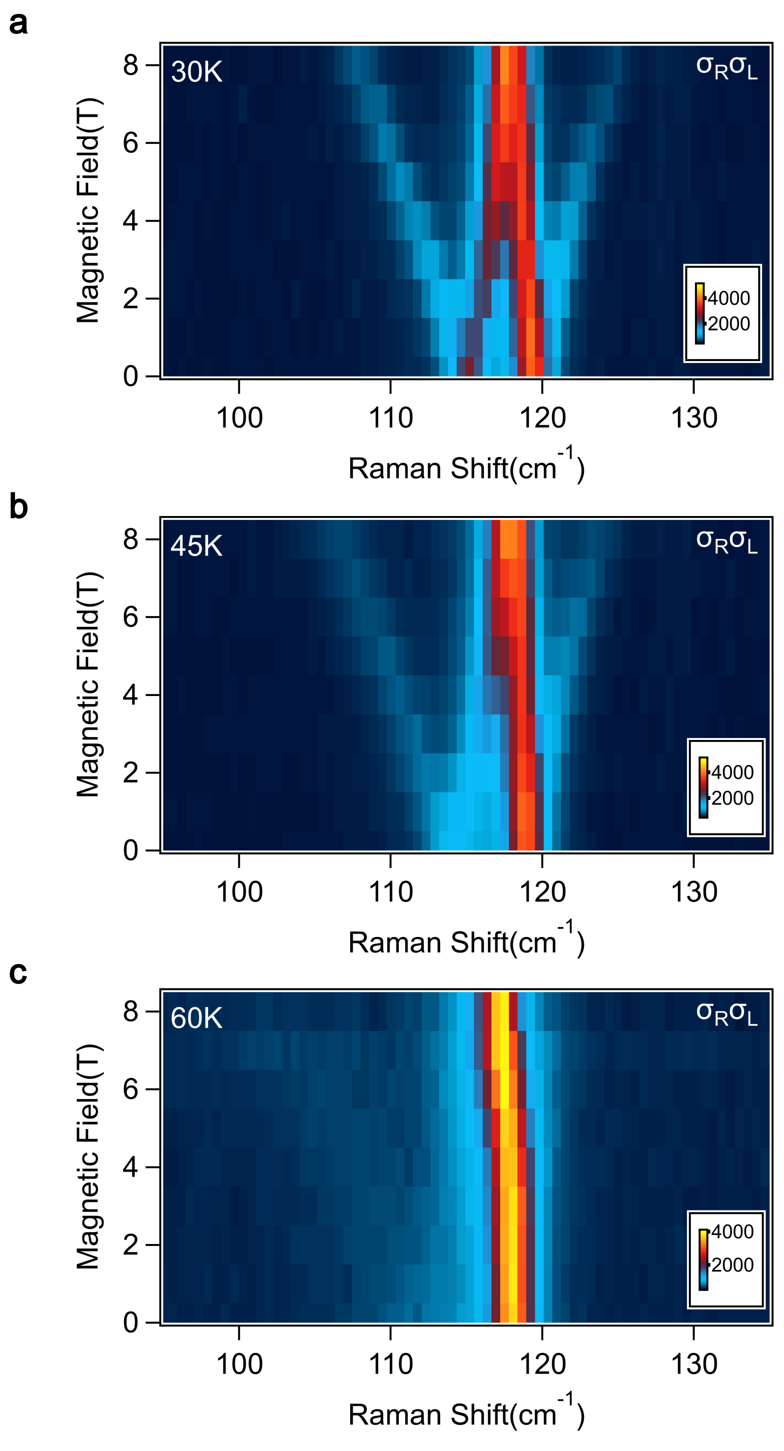

FIG. S6. **Magneto-Raman spectra of magnon polarons in $FePSe_3$ at 30 K, 45 K, and 60 K.** **a**, 30 K. **b**, 45 K. **c**, 60 K. The thickness of the $FePSe_3$ flake is 70 nm. As the temperature increases, the AFM magnons exhibit a redshift, which enlarges the magnon-phonon detunings.

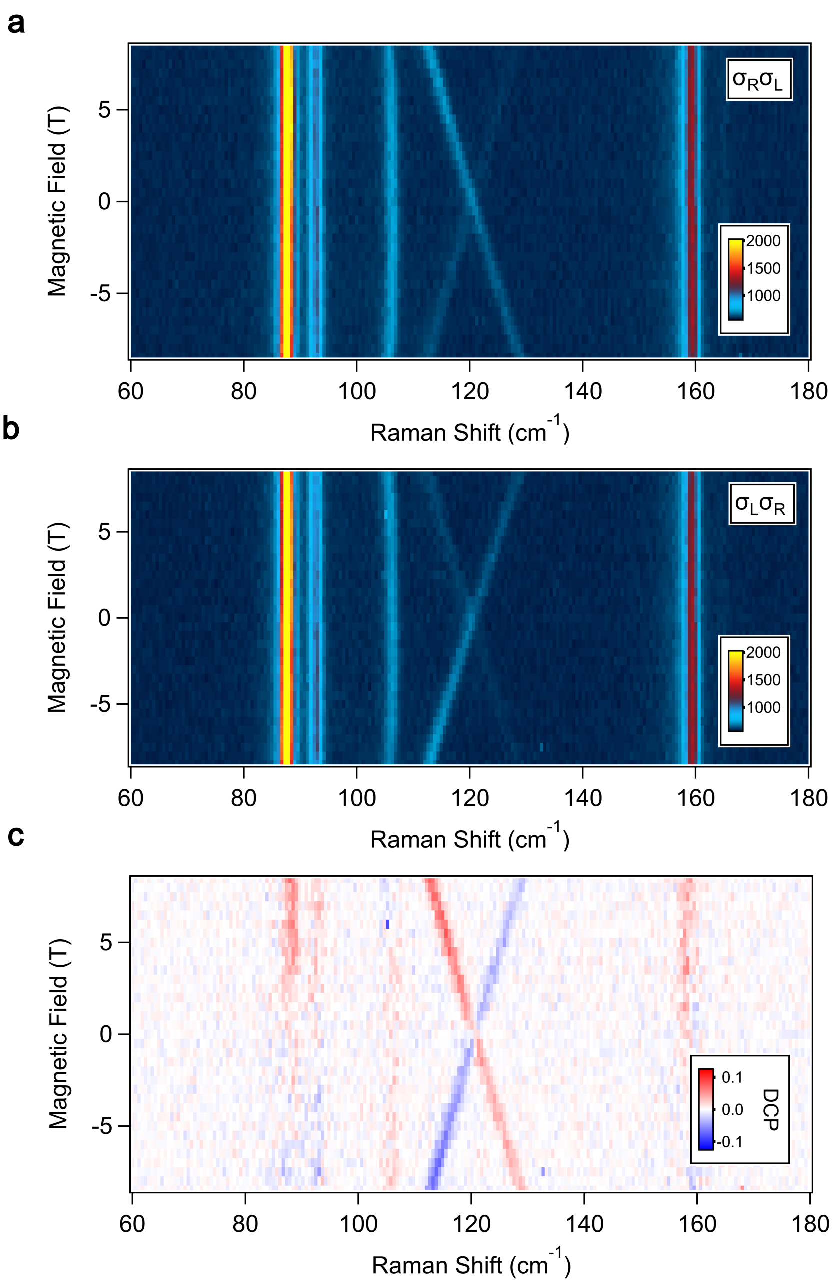

FIG. S7. **Circular polarization resolved magneto-Raman spectra of AFM magnons in $FePS_3$.** $FePS_3$ exhibits a similar Ising-type zigzag spin order as $FePSe_3$ and has a pair of AFM magnon at 121 cm^{-1} at zero magnetic fields. **a**, Magneto-Raman spectra (from 9 T to -9 T) in the $\sigma_R\sigma_L$ channel, and **b**, the $\sigma_L\sigma_R$ channel. **c**, Degree of circular polarization of Raman spectra, defined as $((\sigma_R\sigma_L - \sigma_L\sigma_R)/(\sigma_R\sigma_L + \sigma_L\sigma_R))$. The two Zeeman-split AFM magnons exhibit opposite circular polarization, due to their opposite spin angular momenta.

SUPPLEMENTARY TEXT

S1. Fitting of strongly coupled magnons and phonons. The linear hybridization of magnons and phonons can be described with a phenomenological coupling model (which is derived starting from a microscopic description in the following sections). The energies of the coupled normal modes can be obtained by diagonalizing the matrix M, defined by the relation

$$\begin{pmatrix} \Psi_1 \\ \Psi_2 \\ \Psi_3 \\ \Psi_4 \end{pmatrix} = M \begin{pmatrix} \Phi_{\mathrm{P}_1} \\ \Phi_{\mathrm{P}_2} \\ \Phi_{\mathrm{M}_1} \\ \Phi_{\mathrm{M}_2} \end{pmatrix} \tag{S1}$$

Here Φ_α denotes the uncoupled phonon/magnon wavefunctions, while Ψ_i represent the wavefunctions of the hybridized normal modes, i.e. the magnon polarons (MPs). Assuming the selective hybridization scenario, i.e., each phonon mode only couples to one magnon branch, the coupling matrix M can be written as

$$M = \begin{pmatrix} \omega_{\mathrm{P}_1} & 0 & g_1 & 0 \\ 0 & \omega_{\mathrm{P}_2} & 0 & g_2 \\ g_1 & 0 & \omega_{\mathrm{M}_1} + g\mu_B B & 0 \\ 0 & g_2 & 0 & \omega_{\mathrm{M}_2} - g\mu_B B \end{pmatrix}, \tag{S2}$$

where ω_{P_1}, ω_{P_2}, ω_{M_1} and ω_{M_2} represent the uncoupled frequencies of phonons and magnons. The corresponding coupling strength between each phonon-magnon pair is g_1 and g_2.

S2. General Hamiltonian. The magnon-polaron Hamiltonian can be derived by decomposing the total spin-phonon Hamiltonian as $H = H_{\text{m}} + H_{\text{ph}} + H_{\text{m-ph}}$, with

$$
\begin{aligned}
H_{\text{m}} &= \sum_{\langle ij \rangle} \mathbf{S}_i \cdot (\mathbf{J}_{ij}\mathbf{S}_j) - \Delta \sum_i (S_i^z)^2 \\
H_{\text{ph}} &= \sum_{ij} \left[\frac{\mathbf{p}_i^2}{2M_i} + \frac{k_{ij}}{2}\mathbf{u}_{ij}^2 \right] \\
H_{\text{m}-\text{ph}} &= -\frac{1}{R^2} \sum_{\langle ij \rangle} (\mathbf{u}_{ij} \cdot \mathbf{R}_{ij}) \mathbf{S}_i \cdot ([\boldsymbol{\alpha}\mathbf{J}]_{ij}\mathbf{S}_j).
\end{aligned}
\tag{S3}
$$

where the matrix $\mathbf{J}_{ij}$ encodes the coupling of spins $\mathbf{S}_i$ and $\mathbf{S}_j$, and Δ is a single ion anisotropy. Further, $\mathbf{p}_i$ is the momentum of an ion of mass M_i, $\mathbf{u}_i$ is the ionic displacement from equilibrium, $\mathbf{u}_{ij} = \mathbf{u}_j - \mathbf{u}_i$, and k_{ij} is the elastic tensor. The matrix $\boldsymbol{\alpha}_{ij}$ is proportional to the derivative of $\mathbf{J}_{ij}$ with respect to $\mathbf{u}_i$, and arises for a first order expansion of $\mathbf{J}_{ij}(\mathbf{u}_{ij})$ according to

$$
\mathbf{J}_{ij}(\mathbf{u}_{ij}) \approx \mathbf{J}_{ij} \left(1 - \boldsymbol{\alpha}_{ij} \frac{(\mathbf{u}_{ij} \cdot \mathbf{R}_{ij})}{R_{ij}^2} \right). \tag{S4}
$$

The equilibrium lattice vectors are denoted by $\mathbf{R}_{ij} = \mathbf{R}_i - \mathbf{R}_j$, where $\mathbf{R}_i$ is the equilibrium position of ion i, and $R = |\mathbf{R}_{ij}|$. The formation of MPs requires a linear magnon-phonon coupling, which in $FePSe_3$ arises from anisotropy terms like $J_{xz}S_i^x S_j^z$ and $J_{yz}S_i^y S_j^z$ since the spins point along the z-axis.

Magnetic anisotropies can arise from a number of different mechanisms, and one possibility are the dipole-dipole interactions typically described by the Hamiltonian

$$
H_{\text{d-d}} = \sum_{ij} \left[D_{ij} \mathbf{S}_i \cdot \mathbf{S}_j - 3(\mathbf{S}_i \cdot \mathbf{R}_{ij})(\mathbf{S}_j \cdot \mathbf{R}_{ij}) \right]. \tag{S5}
$$

However, since the magnetic dipole terms have the form $(\mathbf{S}_i \cdot \mathbf{R}_{ij})(\mathbf{S}_j \cdot \mathbf{R}_{ij})$, where $\mathbf{R}_{ij}$ lies in the xy-plane, this interaction cannot give rise to terms like J_{xz} and J_{yz}. Another possibility is a spin-orbit coupling (SOC) mediated exchange or coupling to the crystal field. The anisotropic terms need to be consistent with the magnetic point group symmetries.

S3. Hamiltonian parameters from first principles. To parameterize the Hamiltonian of Eq. S3 we performed first principles calculations with the ABINIT electronic structure code.

Parameter	J_1^F	J_1^A	J_2^F	J_2^A	J_3	Δ
Value (meV)	3.35	2.30	−0.1	−0.65	−0.35	2.33

TABLE S1. **Magnetic parameters of $FePSe_3$.** Magnetic parameters of $FePSe_3$ calculated from first principles.

We employed the DFT+U formalism using the local density approximation with projector augmented wave (PAW) pseudopotentials, a plane wave cut-off of 20 Ha and 40 Ha for the plane wave and PAW part, and included an empirical Hubbard U and Hund's J of 3.5 eV and 0.25 eV calculated with the OCTOPUS code via the ACBN0 functional. A Γ-centered Monkhorst-Pack grid with dimensions $8 \times 6 \times 8$ was used to sample the Brillouin zone.

The magnetic ground state was found to have a zig-zag antiferromagnetic order with magnetic moments 3.22 μ_B mainly aligned along the z-axis. The DFT+U wave functions were mapped onto effective localized orbitals via the WANNIER90 code, and the magnetic parameters were calculated by the PYTHON package TB2J relying on the magnetic force theorem. The phonon frequencies and polarization vectors at the Γ point were calculated using density functional perturbation theory (DFPT) as implemented in ABINIT, assuming a ferromagnetic interlayer coupling. The atomic positions and stresses were relaxed to below 10^{-6} Ha/Bohr. The calculated phonon modes are in good agreement with previous DFT calculations and Raman scattering data. The magnon-phonon couplings were found by comparing the magnetic parameters calculated in the equilibrium state and for ionic coordinates displaced according to the phonon polarizations.

S4. Magnon bandstructure and phonon modes. The magnon Hamiltonian H_m is diagonalized by a Holstein-Primakoff transformation followed by a Bogoliubov transform. Taking into account the zigzag antiferromagnetic order, H_m has the structure

$$H_\mathrm{m} = \sum_{\mathbf{k}} \Phi_{\mathbf{k}}^\dagger H_{\mathbf{k}} \Phi_{\mathbf{k}}, \tag{S6}$$

where the Nambu spinor is $\Phi_{\mathbf{k}}^\dagger = (a_{1\mathbf{k}}^\dagger, a_{2\mathbf{k}}^\dagger, b_{1,-\mathbf{k}}, b_{2,-\mathbf{k}})$, and $a_{i\mathbf{k}}$ and $b_{i\mathbf{k}}$ act on the sublattices

of spins pointing up and down, respectively. The Hamiltonian $H_{\mathbf{k}}$ can be written as

$$H_{\mathbf{k}} = \begin{pmatrix} A & B^* & C & D^* \\ B & A & D & C \\ C & D^* & A & B^* \\ D & C & B & A \end{pmatrix}, \tag{S7}$$

where the matrix elements are given in terms of the magnetic parameters by

$$\begin{aligned} A_{\mathbf{k}} &= J_1^A - 2J_1^F + 2J_2^F[\cos(\mathbf{k}\cdot\mathbf{b}_1) - 1] + 4J_2^A + 3J_3 - \Delta \\ B_{\mathbf{k}} &= J_1^F(e^{-i\mathbf{k}\cdot\mathbf{a}_2} + e^{-i\mathbf{k}\cdot\mathbf{a}_3}) \\ C_{\mathbf{k}} &= 2J_2^A[\cos(\mathbf{k}\cdot\mathbf{b}_2) + \cos(\mathbf{k}\cdot\mathbf{b}_3)] \\ D_{\mathbf{k}} &= J_1^A e^{-i\mathbf{k}\cdot\mathbf{a}_1} + J_3(e^{-i\mathbf{k}\cdot\mathbf{c}_1} + e^{-i\mathbf{k}\cdot\mathbf{c}_2} + e^{-i\mathbf{k}\cdot\mathbf{c}_3}). \end{aligned}$$

Here J_i^F (J_i^A) is the interaction between ith nearest neighbors with a ferromagnetic (antiferromagnetic) alignment, and $\mathbf{a}_i$, $\mathbf{b}_i$ and $\mathbf{c}_i$ are respectively the nearest, next nearest and third nearest neighbor lattice vectors. Diagonalizing the system via a Bogoliubov transform, the magnon Hamiltonian and energies are given by[1]

$$\begin{aligned} H_{\mathrm{m}} &= \sum_{\lambda\mathbf{k}} \epsilon_{\lambda\mathbf{k}} \left[\alpha^\dagger_{\lambda\mathbf{k}}\alpha_{\lambda\mathbf{k}} + \beta^\dagger_{\lambda,-\mathbf{k}}\beta_{\lambda,-\mathbf{k}}\right] \\ \epsilon_{\pm,\mathbf{k}} &= \left(A^2 + |B|^2 - C^2 - |D|^2 \pm \sqrt{4|AB^* - CD^*|^2 - |BD^* - DB^*|^2}\right)^{1/2}. \end{aligned} \tag{S8}$$

The magnetic parameters found from our first principles calculations are given in Tab. S1, and the corresponding magnon bandstructure is shown in Fig. S11. For the parameters of Tab. S1, the energy of the lower magnon branch at Γ is $\epsilon_- = 13.86$ meV.

To diagonalize the bare phonon Hamiltonian H_{ph} the displacement operators $\mathbf{u}_i$ can be expanded in normal modes like

$$\mathbf{u}_i^m = \sum_{\lambda\mathbf{q}} \hat{\mathbf{e}}^m_{\lambda\mathbf{q}} u_{\lambda\mathbf{q}} e^{i\mathbf{q}\cdot\mathbf{R}_i}, \tag{S9}$$

where we have introduced the label m to distinguish inequivalent ions within the primitive unit cell, and $\hat{\mathbf{e}}^m_{\lambda\mathbf{q}}$ is a unit vector describing the phonon polarization of mode λ at wave

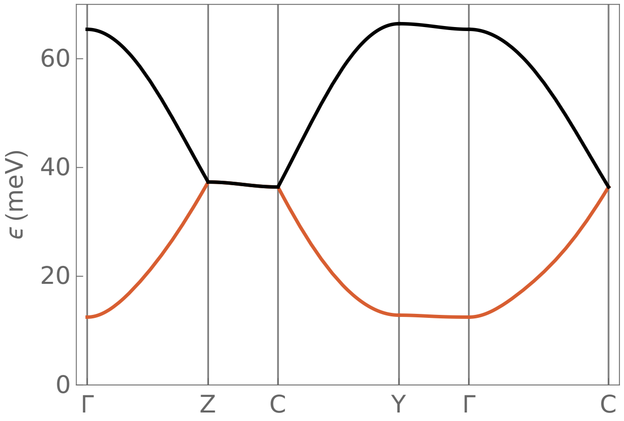

FIG. S8. **Magnon bandstructure of $FePSe_3$.** Magnon bandstructure of $FePSe_3$ obtained from Eq. S8 with the magnetic parameters of Tab. S1.

Phonon mode	1	2	3	4
ω (cm^{-1})	109.5	111.6	112.1	115.6
ω (meV)	13.67	13.83	13.89	14.33
Irreducible representation	A_g	B_g	B_g	A_g
$x^{(0)}$ (Å)	0.1312	0.1304	0.1301	0.1281

TABLE S2. **Phonon energies of $FePSe_3$.** Calculated energy and irreducible representation of the C_{2h} point group of the Raman active phonon modes of $FePSe_3$ proximate to the lower magnon branch at 110.8 cm^{-1}.

vector $\mathbf{q}$. A similar expansion can be done for the momentum operators. The final form of the Hamiltonian is obtained by writing $u_{\lambda\mathbf{q}} = x^{(0)}_{\lambda\mathbf{q}}(\gamma^\dagger_{\lambda\mathbf{q}} + \gamma_{\lambda\mathbf{q}})$[2], where $x^{(0)}_{\lambda\mathbf{q}} = (\hbar/2M\omega_{\lambda\mathbf{q}})^{1/2}$ is the single phonon amplitude. This gives

$$H_{\text{ph}} = \sum_{\lambda\mathbf{k}} \omega_{\lambda\mathbf{k}} \gamma^\dagger_{\lambda\mathbf{k}} \gamma_{\lambda\mathbf{k}}. \tag{S10}$$

The phonon energies and irreducible representations of the Raman modes proximate to the lower magnon branch are given in Tab. S2. The corresponding phonon displacement patterns are illustrated in Fig. S9.

S5. Magnon-phonon coupling. A linear magnon-phonon coupling arises from magnetic anisotropy terms like $S^x_i S^z_j$ and $S^y_i S^z_j$. The most general Hamiltonian involving such terms

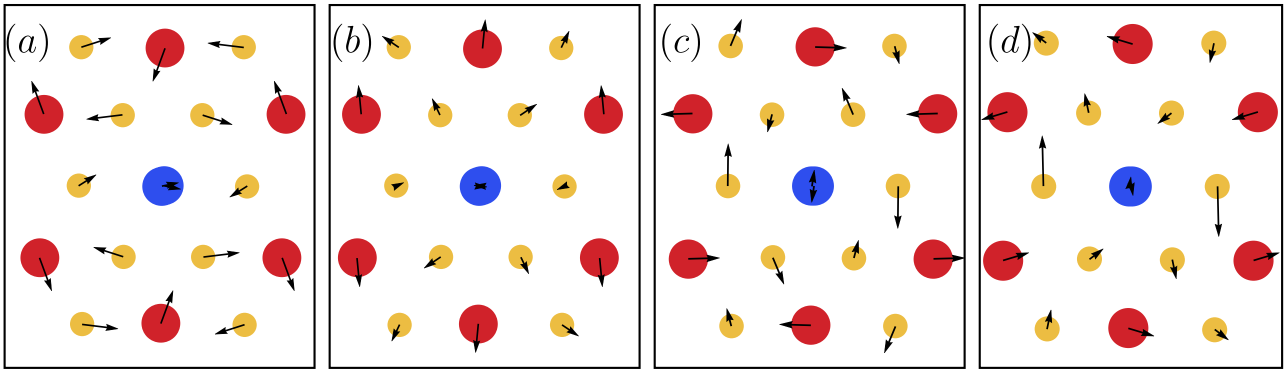

FIG. S9. **Phonon displacement patterns of FePSe$_3$.** Displacement patterns of the phonon modes 1 through 4 given in Table S2.

is

$$H_{\text{m-ph}} = -\frac{\alpha}{R^2}\sum_{\langle ij\rangle}(\mathbf{u}_j - \mathbf{u}_i)\cdot(\mathbf{R}_j - \mathbf{R}_i) \times \Big[J_{xz}\big(S_i^x S_j^z + S_i^z S_j^x\big) + J_{yz}\big(S_i^y S_j^z + S_i^z S_j^y\big)\Big]. \tag{S11}$$

The explicit expressions for the spins in terms of bosonic operators differ depending on the bond, and can be written as

$$J_{xz}\big(S_i^x S_j^z + S_i^z S_j^x\big) + J_{yz}\big(S_i^y S_j^z + S_i^z S_j^y\big)$$

$$= (\uparrow\uparrow) = \sqrt{\frac{S^3}{2}}\Big[J_{xz}(a_i^\dagger + a_i + a_j^\dagger + a_j) + iJ_{yz}(a_i^\dagger - a_i + a_j^\dagger - a_j)\Big] \tag{S12}$$

$$= (\downarrow\downarrow) = -\sqrt{\frac{S^3}{2}}\Big[J_{xz}(b_i^\dagger + b_i + b_j^\dagger + b_j) + iJ_{yz}(b_i^\dagger - b_i + b_j^\dagger - b_j)\Big] \tag{S13}$$

$$= (\uparrow\downarrow) = \sqrt{\frac{S^3}{2}}\Big[J_{xz}(b_j^\dagger + b_j - a_i^\dagger - a_i) + iJ_{yz}(b_j^\dagger - b_j - a_i^\dagger + a_i)\Big] \tag{S14}$$

$$= (\downarrow\uparrow) = \sqrt{\frac{S^3}{2}}\Big[J_{xz}(b_i^\dagger + b_i - a_j^\dagger - a_j) + iJ_{yz}(b_i^\dagger - b_i - a_j^\dagger + a_j)\Big], \tag{S15}$$

where the arrows in parentheses indicate the direction (up or down) of the spins on sites (ij).

To evaluate the magnon-phonon coupling, it is thus enough to consider products of the form

$$H^{ij}_{\text{m-ph}} = \sum_i \big(a_i^\dagger \pm a_i\big)(\mathbf{u}_j - \mathbf{u}_i)\cdot(\mathbf{R}_j - \mathbf{R}_i).$$
$$\rightarrow \sum_\lambda u_{\lambda\mathbf{q}}(a_{\mathbf{k}}^\dagger \pm a_{\mathbf{k}})(\hat{\mathbf{e}}^i_{\lambda\mathbf{q}} - \hat{\mathbf{e}}^j_{\lambda\mathbf{q}})\cdot(\mathbf{R}_j - \mathbf{R}_i) \tag{S16}$$

where the last expression holds in the long wavelength limit $\mathbf{k},\mathbf{q}\rightarrow 0$, and $\hat{\mathbf{e}}^i_{\lambda\mathbf{q}}$ is a unit vector giving the phonon polarization. Adding up the contributions from all spins in the unit cell, we find the Hamiltonian

$$H_{\text{m-ph}} = -\sum_\lambda u_\lambda\big[(\Gamma_{\lambda 1}a_1^\dagger + \Gamma^*_{\lambda 1}a_1) + (\Gamma_{\lambda 2}b_1^\dagger + \Gamma^*_{\lambda 2}b_1)$$
$$+ (\Gamma_{\lambda 3}b_2^\dagger + \Gamma^*_{\lambda 3}b_2) + (\Gamma_{\lambda 4}a_2^\dagger + \Gamma^*_{\lambda 4}a_2)\big] \tag{S17}$$

where the index i now labels the spin raising and lowering operators at $\mathbf{k}=0$ and at site i in the unit cell. The magnon-phonon coupling constants are given by

$$\Gamma^x_1 = \frac{\sqrt{8S^3}}{R^2}J_{xz}\big[\alpha_1\hat{\mathbf{e}}_{12}\cdot\mathbf{a}_1 - \hat{\mathbf{e}}_{14}\cdot(\alpha_2\mathbf{a}_2 + \alpha_3\mathbf{a}_3)\big] \tag{S18}$$
$$\Gamma^x_2 = -\frac{\sqrt{8S^3}}{R^2}J_{xz}\big[\alpha_1\hat{\mathbf{e}}_{21}\cdot\mathbf{a}_1 + \hat{\mathbf{e}}_{23}\cdot(\alpha_2\mathbf{a}_2 + \alpha_3\mathbf{a}_3)\big]$$
$$\Gamma^x_3 = \frac{\sqrt{8S^3}}{R^2}J_{xz}\big[\alpha_1'\hat{\mathbf{e}}_{34}\cdot\mathbf{a}_1 + \hat{\mathbf{e}}_{32}\cdot(\alpha_2\mathbf{a}_2 + \alpha_3\mathbf{a}_3)\big]$$
$$\Gamma^x_4 = -\frac{\sqrt{8S^3}}{R^2}J_{xz}\big[\alpha_1'\hat{\mathbf{e}}_{43}\cdot\mathbf{a}_1 - \hat{\mathbf{e}}_{41}\cdot(\alpha_2\mathbf{a}_2 + \alpha_3\mathbf{a}_3)\big]$$
$$\Gamma^y_1 = i\frac{\sqrt{8S^3}}{R^2}J_{yz}\big[\alpha_1\hat{\mathbf{e}}_{12}\cdot\mathbf{a}_1 - \hat{\mathbf{e}}_{14}\cdot(\alpha_2\mathbf{a}_2 + \alpha_3\mathbf{a}_3)\big]$$
$$\Gamma^y_2 = -i\frac{\sqrt{8S^3}}{R^2}J_{yz}\big[\alpha_1\hat{\mathbf{e}}_{21}\cdot\mathbf{a}_1 + \hat{\mathbf{e}}_{23}\cdot(\alpha_2\mathbf{a}_2 + \alpha_3\mathbf{a}_3)\big]$$
$$\Gamma^y_3 = i\frac{\sqrt{8S^3}}{R^2}J_{yz}\big[\alpha_1'\hat{\mathbf{e}}_{34}\cdot\mathbf{a}_1 + \hat{\mathbf{e}}_{32}\cdot(\alpha_2\mathbf{a}_2 + \alpha_3\mathbf{a}_3)\big]$$
$$\Gamma^y_4 = -i\frac{\sqrt{8S^3}}{R^2}J_{yz}\big[\alpha_1'\hat{\mathbf{e}}_{43}\cdot\mathbf{a}_1 - \hat{\mathbf{e}}_{41}\cdot(\alpha_2\mathbf{a}_2 + \alpha_3\mathbf{a}_3)\big].$$

Making a rotation to the eigenbasis of the magnon operators, the Hamiltonian takes

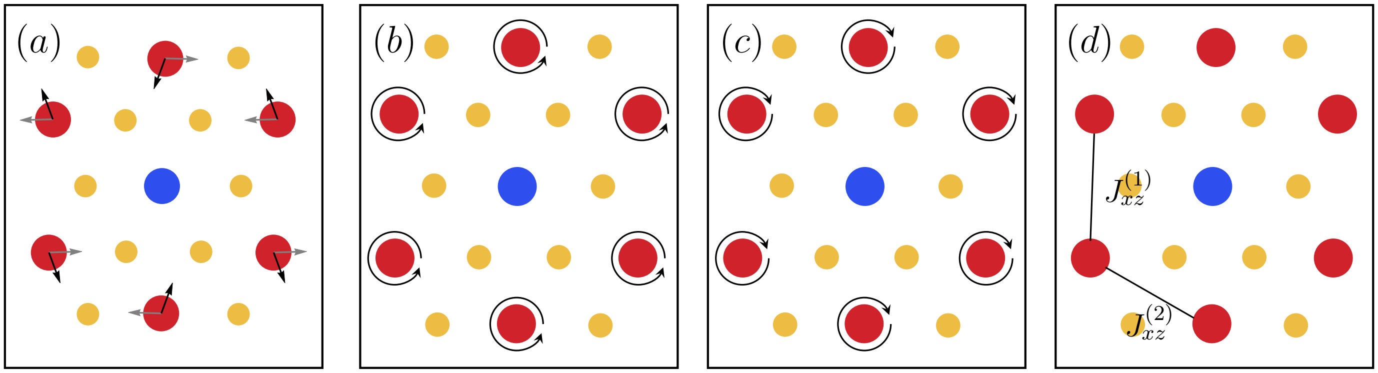

FIG. S10. **Magnon-induced circular phonons modes of $FePSe_3$.** Displacement patterns of the phonon modes 1 through 4 given in Table S2, whose energies are proximate to the lower magnon branch of $FePSe_3$.

the form

$$H_{\text{m-ph}} = -\sum_{\lambda} u_\lambda [(\Gamma_{\lambda\alpha}\alpha^\dagger + \Gamma^*_{\lambda\alpha}\alpha) + (\Gamma_{\lambda\beta}\beta^\dagger + \Gamma^*_{\lambda\beta}\beta)].$$

The final expressions for the magnon-phonon coupling constants are then

$$\begin{aligned}\Gamma_\alpha = \frac{\sqrt{8S^3}}{R^2} J_{xz} \big[&(U\hat{\mathbf{e}}_{14} + V\hat{\mathbf{e}}_{23}) \cdot (\alpha_2 \mathbf{a}_2 + \alpha_3 \mathbf{a}_3) \\ &+ (U - V)(\alpha_1 \hat{\mathbf{e}}_{12} + \alpha_1' \hat{\mathbf{e}}_{34}) \cdot \mathbf{a}_1 \big] \\ + i \frac{\sqrt{8S^3}}{R^2} J_{yz} \big[&(U\hat{\mathbf{e}}_{14} + V\hat{\mathbf{e}}_{23}) \cdot (\alpha_2 \mathbf{a}_2 + \alpha_3 \mathbf{a}_3) \\ &+ (U - V)(\alpha_1 \hat{\mathbf{e}}_{12} + \alpha_1' \hat{\mathbf{e}}_{34}) \cdot \mathbf{a}_1 \big]\end{aligned} \tag{S19}$$

$$\begin{aligned}\Gamma_\beta = -\frac{\sqrt{8S^3}}{R^2} J_{xz} \big[&(V\hat{\mathbf{e}}_{14} + U\hat{\mathbf{e}}_{23}) \cdot (\alpha_2 \mathbf{a}_2 + \alpha_3 \mathbf{a}_3) \\ &+ (V - U)(\alpha_1 \hat{\mathbf{e}}_{12} + \alpha_1' \hat{\mathbf{e}}_{34}) \cdot \mathbf{a}_1 \big] \\ + i \frac{\sqrt{8S^3}}{R^2} J_{yz} \big[&(V\hat{\mathbf{e}}_{14} + U\hat{\mathbf{e}}_{23}) \cdot (\alpha_2 \mathbf{a}_2 + \alpha_3 \mathbf{a}_3) \\ &+ (V - U)(\alpha_1 \hat{\mathbf{e}}_{12} + \alpha_1' \hat{\mathbf{e}}_{34}) \cdot \mathbf{a}_1 \big].\end{aligned} \tag{S20}$$

Here, the constants U and V are defined via the magnon transformation $a_i \approx U\alpha - V\beta^\dagger$ and $b_i^\dagger \approx V\alpha - U\beta^\dagger$, and numerically we find $U = 0.72$ and $V = -0.14$ from the diagonalization of Eq. S8.

S6. Magnon-polaron Hamiltonian. To use the expressions in Eq. S19 to calculate the magnon-phonon coupling of $FePSe_3$, we need to find the parameters $\alpha = [(J_{xz}(0) -$

$J_{xz}(d))/J_{xz}(0)](d/R)$. Here, the magnetic anisotropies J_{xz} and J_{yz} are evaluated in the equilibrium state and in the phonon modulated states where the Fe-Fe bond distance has changed by d. In addition, we need the single phonon amplitudes $x_\lambda^{(0)}$ (see Tab. S2). The magnetic anisotropies are given in Tabs. S3 and S4, and the Fe-Fe distances are shown in Tab. S5.

Calculating the magnon-phonon coupling for the phonon modes 1 through 4, we find the values of Tab. S6. As can be clearly seen, the coupling to phonon modes 2 and 4 is strongly suppressed. This can be qualitatively understood by noting that these modes correspond to a rigid motion of FM chains of $FePSe_3$ (see Fig. S9). Since the magnon modes at $\mathbf{k} = 0$ are mainly localized either in the upper or lower FM chain, the magnon-phonon coupling is thus quite insensitive to changes in the interchain distance. We assume in the following that the coupling to these modes vanishes.

For phonon modes 1 and 3, we find the former mainly couples to the magnons via a modulation of J_{xz}, while the latter mainly couples via a modulation of J_{yz}. Consequently, the couplings are given by $\Gamma_{\alpha,1} = \Gamma_{\beta,1} = g_1 \approx 0.27$, and $\Gamma_{\alpha,3} = -\Gamma_{\beta,3} = g_3 \approx 0.25i$. The resulting magnon-polaron Hamiltonian, written in the basis $\Psi = [\alpha, \beta, \gamma_1, \gamma_3]^T$, has the form

$$H = \Psi^\dagger \begin{pmatrix} \epsilon_\alpha & 0 & g_1 & ig_3 \\ 0 & \epsilon_\beta & g_1 & -ig_3 \\ g_1 & g_1 & \omega_1 & 0 \\ -ig_3 & ig_3 & 0 & \omega_3 \end{pmatrix} \Psi. \tag{S21}$$

To get some intuition for the coupled system, we note that for $\omega_1 = \omega_3$ and $g_1 = g_3 = g$ the Hamiltonian can be rewritten using the transformation $\gamma_\pm = (g\gamma_1 \pm ig\gamma_3)/\sqrt{2g^2} = (\gamma_1 \pm i\gamma_3)/\sqrt{2}$ to give

$$H = \Psi^\dagger \begin{pmatrix} \epsilon_\alpha & 0 & \sqrt{2}g & 0 \\ 0 & \epsilon_\beta & 0 & \sqrt{2}g \\ \sqrt{2}g & 0 & \omega & 0 \\ 0 & \sqrt{2}g & 0 & \omega \end{pmatrix} \Psi. \tag{S22}$$

This Hamiltonian shows the selective coupling between the magnon modes and the circular phonon modes $\gamma_\pm$ (this terminology will be explained in the next section) shown in

	$J_{xz}^{(1)}$	$J_{xz}^{(2)}$	$J_{xz}^{(3)}$	$J_{xz}^{(4)}$	$J_{xz}^{(5)}$	$J_{xz}^{(6)}$
Equil.	0.001	−0.001	0.001	0.001	−0.001	0.001
Mode 1	0.0	−0.005	−0.005	0.0	−0.005	−0.005
Mode 2	−0.005	−0.004	−0.012	−0.008	−0.012	−0.004
Mode 3	−0.006	0.001	−0.002	0.002	−0.002	0.001
Mode 4	0.001	−0.004	−0.004	0.001	−0.004	−0.004

TABLE S3. **Magnetic parameters of $FePSe_3$.** Magnetic anisotropy parameters $J_{xz}^{(i)}$ calculated from first principles labeled according to Fig. S10(d).

	$J_{yz}^{(1)}$	$J_{yz}^{(2)}$	$J_{yz}^{(3)}$	$J_{yz}^{(4)}$	$J_{yz}^{(5)}$	$J_{yz}^{(6)}$
Equil.	0.0	−0.018	0.018	0.0	0.018	−0.018
Mode 1	−0.002	−0.013	0.013	0.002	0.013	−0.013
Mode 2	−0.001	−0.001	0.008	0.003	0.008	−0.001
Mode 3	−0.004	−0.005	−0.001	−0.004	−0.001	−0.005
Mode 4	−0.005	0.025	−0.025	0.005	−0.025	0.025

TABLE S4. **Magnetic parameters of $FePSe_3$.** Magnetic anisotropy parameters $J_{yz}^{(i)}$ calculated from first principles labeled according to Fig. S10(d).

Fig. S10. Since in practice $\omega_1 \approx \omega_3$ and $g_1 \approx g_3$, we expect the circular basis to give a good qualitative understanding of the physics of the coupled system.

S7. Magnon-induced chiral phonons. The spin angular momentum of the magnons along the z-direction is obtained from the operator $S^z = \sum_i S_i^z$. In the Holstein-Primakoff basis this operator is given by $S^z = \sum_i(-a_i^\dagger a_i + b_i^\dagger b_i)$, or $S^z = -\alpha^\dagger\alpha + \beta^\dagger\beta$. Thus, the magnons at Γ have angular momentum $S^z = \mp 1$ in the α/β-branch, respectively.

To understand the selective magnon-phonon coupling discussed in the previous section,

	$d^{(1)}$	$d^{(2)}$	$d^{(3)}$	$d^{(4)}$	$d^{(5)}$	$d^{(6)}$
Equil.	3.657	3.491	3.491	3.657	3.491	3.491
Mode 1	3.729	3.456	3.456	3.729	3.456	3.456
Mode 2	3.783	3.484	3.497	3.530	3.497	3.484
Mode 3	3.651	3.422	3.559	3.664	3.559	3.422
Mode 4	3.639	3.499	3.499	3.639	3.499	3.499

TABLE S5. **Phonon modulated Fe-Fe distances in $FePSe_3$.** Modulated Fe-Fe distances for the phonon modes shown in Fig. S9. The distances are labeled according to Fig. S10(d).

	Mode 1	Mode 2	Mode 3	Mode 4
Γ_α (meV)	0.27	$-0.01 + 0.01i$	$0.01 + 0.25i$	-0.03
Γ_β (meV)	0.27	$-0.01 - 0.01i$	$0.01 - 0.25i$	-0.03

TABLE S6. **Magnetic parameters of FePSe$_3$.** Magnetic anisotropy parameters $J_{yz}^{(i)}$ calculated from first principles labeled according to Fig. S10(d).

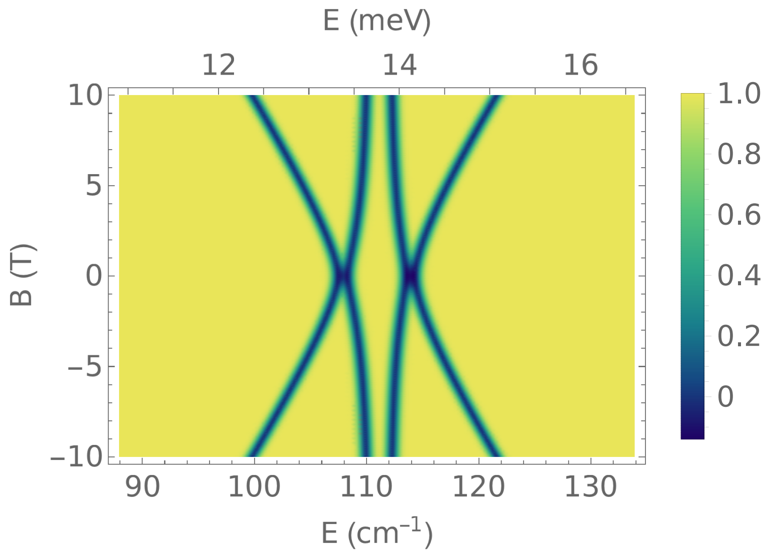

FIG. S11. **Coherent magnon-phonon coupling in FePSe$_3$.** Magnon-polaron energies of FePSe$_3$ as a function of magnetic field B, obtained from Eq. S21 with parameters from Tabs. S2 and S6.

we follow the discussion in Ref.[3] and define the phonon angular momentum as

$$\mathbf{L} = \sum_{i\alpha} M_{i\alpha} \mathbf{u}_{i\alpha} \times \dot{\mathbf{u}}_{i\alpha}, \tag{S23}$$

where $M_{i\alpha}$ is the mass of atom α in unit cell i. In equilibrium the z-component of phonon angular momentum can be written

$$L^z = \sum_{n\mathbf{k}} \left[f(\omega_{n\mathbf{k}}) + \frac{1}{2} \right] l^z_{n\mathbf{k}}, \tag{S24}$$

where $f(\omega_{n\mathbf{k}})$ is the Bose-Einstein distribution and $l^z_{n\mathbf{k}}$ is the angular momentum of phonon mode n at wave vector $\mathbf{k}$. The angular momentum of a given phonon mode is given by

$$l^z_{n\mathbf{k}} = \hbar \epsilon^\dagger_{n\mathbf{k}} M \epsilon_{n\mathbf{k}}. \tag{S25}$$

Here $\epsilon_{n\mathbf{k}}^{\dagger} = (\epsilon_{n\mathbf{k}1}^{x}, \epsilon_{n\mathbf{k}1}^{y}, \ldots, \epsilon_{n\mathbf{k}m}^{x}, \epsilon_{n\mathbf{k}m}^{y})$ is an in-plane polarization vector containing the displacements of all m atoms in the unit cell, and $M = \sigma_y \otimes I_m$ is the product of the Pauli matrix σ_y and the identity matrix of order m.

We note that for real $\epsilon_{n\mathbf{k}}$ the angular momentum vanishes, as expected for a linearly polarized motion. For the circular phonon modes $\gamma_{\pm}$ defined in the previous section, the polarization vector for each Fe atom in the unit cell can be written as $\boldsymbol{\epsilon}_{\pm} = (\hat{\mathbf{e}}_1 \pm i\hat{\mathbf{e}}_3)/\sqrt{2}$, with $\hat{\mathbf{e}}_i$ the polarization of mode i at that atom. Since the phonon modes 1 and 3 are predominantly polarized along the y- and x-directions, respectively (see Fig. S9), the circular phonons have a non-zero angular momentum $L_{\pm}^{z} = \mp l$. Here $l = 4(\hbar/2)\epsilon_{\pm}^{2}$, where the factor four comes from the number of Fe atoms in the unit cell, and $\epsilon_{\pm} = |\boldsymbol{\epsilon}_{\pm}|$. Consequently, we see that the selective magnon-phonon coupling happens between modes with equal directions of angular momentum.

S8. Degree of circular polarization of Raman spectra. The one-magnon and one-phonon Raman scattering Hamiltonian has the general form

$$H_{\mathrm{R}} = \left[R_m(\delta_{s_{\mathrm{in}},L}\delta_{s_{\mathrm{out}},R}\alpha^{\dagger} + \delta_{s_{\mathrm{in}},R}\delta_{s_{\mathrm{out}},L}\beta^{\dagger}) + R_{\gamma_1}\gamma_1^{\dagger} + R_{\gamma_3}\gamma_3^{\dagger} + H.c.\right]a_{s_{\mathrm{out}}}^{\dagger}a_{s_{\mathrm{in}}}, \tag{S26}$$

where the Raman coefficients R_i depend on the microscopic interactions of the system. Transforming the Hamiltonian to the magnon-polaron eigenbasis, we find

$$\begin{aligned} H_{\mathrm{R}} &= \sum_i \left[R_i\eta_i^{\dagger} + H.c.\right]a_{s_{\mathrm{out}}}^{\dagger}a_{s_{\mathrm{in}}} \\ R_i &= R_m(\delta_{s_{\mathrm{in}},L}\delta_{s_{\mathrm{out}},R}U_{\alpha i} + \delta_{s_{\mathrm{in}},R}\delta_{s_{\mathrm{out}},L}U_{\beta i}) + R_{\gamma_1}U_{\gamma_1 i} + R_{\gamma_3}U_{\gamma_3 i}, \end{aligned} \tag{S27}$$

where the new Raman coefficients R_i are expressed in terms of the unitary matrix U that diagonalizes Eq. S21. The Kronecker delta functions encode the angular momentum selection rules of magnon scattering.

We now use this Hamiltonian to calculate the Raman cross-section for circularly polarized incident light. We denote the Raman cross-section for incident polarization s_{in} and

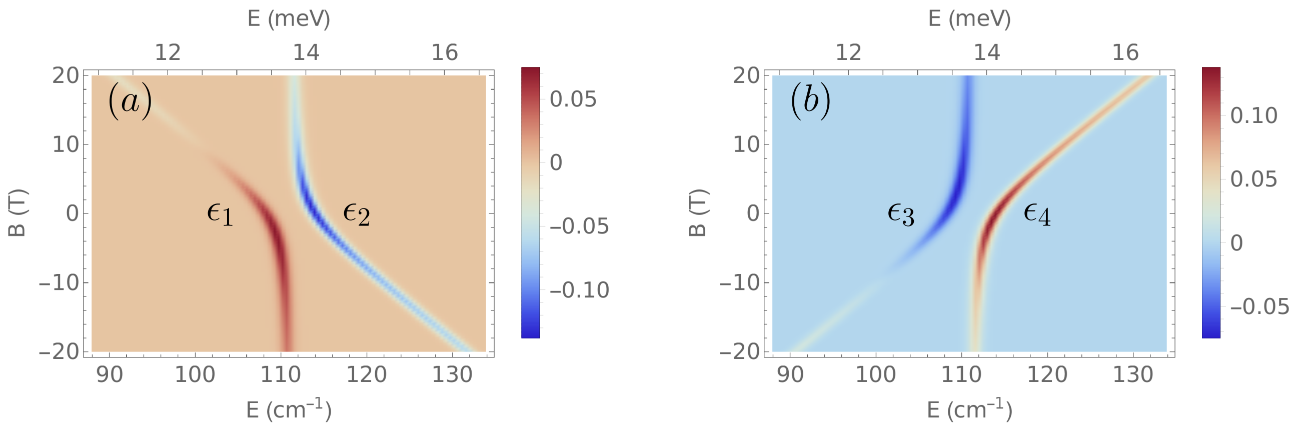

FIG. S12. **Raman circular dichroism of FePSe$_3$.** (a, b) Polarization dependence of magnon polaron states as a function of magnetic field B. The states $|\epsilon_i\rangle$ with energy ϵ_i are obtained by solving Eq. S21 under the assumptions $\epsilon_{\alpha/\beta} = \epsilon \pm B$, $\omega_1 = \omega_3 = \epsilon$ and $g_1 = g_3 = g$.

outgoing polarization s_{out} by

$$\begin{aligned}\sigma_{s_{\text{in}}s_{\text{out}}} &= \sum_i |\langle s_{\text{out}}, \epsilon_i | R_i \eta_i^\dagger a_{s_{\text{out}}}^\dagger a_{s_{\text{in}}} | s_{\text{in}}, 0\rangle|^2 \delta(\omega - \epsilon_i) \\ &= |R_i|^2 \delta(\omega - \epsilon_i).\end{aligned} \tag{S28}$$

Here $|\epsilon_i\rangle$ are eigenstates of Eq. S21 with energy ϵ_i, created by the operators $\eta_i^\dagger$, and $\omega = \omega_{\text{in}} - \omega_{\text{out}}$. From the Raman cross-section we define the degree of circular polarization as

$$C = \frac{\sigma_{\text{LR}} - \sigma_{\text{RL}}}{\sigma_{\text{LR}} + \sigma_{\text{RL}}}, \tag{S29}$$

which is shown in Fig. 5d of the main text. This figure agrees well with the experimental findings.

The degree of circular polarization is determined by a delicate interplay of the coupled magnons and phonons. For large values of R_{γ_1}/R_m the spectrum looks as in Fig. 5d, while for small values of R_{γ_1}/R_m the spectrum looks like that of the uncoupled system. Depending on the sign of the magnon-phonon coupling g, either the lower or upper modes in each magnon polaron pair changes its polarization dependence as a function of the magnetic field B (see Fig. S12a and Fig. S12b). This is due to either a constructive or destructive interference of the magnon and phonon contributions to the Raman coefficients R_i: When the contributions add up the polarization dependence is unchanged as a function of B, while when the contributions partially cancel the polarization dependence changed with B.

We further note that for large B the degree of circular polarization vanishes for the phonon modes, and reverts to the polarization dependence expected for the magnons in the uncoupled limit (see Fig. 5e).

S9. Analytical expression for the degree of circular polarization in the circular phonon limit. The physical picture discussed above is most easily obtained by solving Eq. S21 under the assumption that $\epsilon_{\alpha/\beta} = \epsilon \pm B$, $\omega_1 = \omega_3 = \epsilon$ and $g_1 = g_3 = g$. In this limit, the numerator of the degree of circular polarization can be written analytically as

$$\begin{aligned}\sigma_{\mathrm{LR}} - \sigma_{\mathrm{RL}} = & \frac{|2R_+ - if_+R_m|^2 - |2R_+|^2}{d_+}\delta(\omega - \epsilon_1) \\ & + \frac{|2R_+ - if_-R_m|^2 - |2R_+|^2}{d_-}\delta(\omega - \epsilon_2) \\ & + \frac{|2R_-|^2 - |2R_- - if_-R_m|^2}{d_-}\delta(\omega - \epsilon_3) \\ & + \frac{|2R_-|^2 - |2R_- - if_+R_m|^2}{d_+}\delta(\omega - \epsilon_4)\end{aligned} \tag{S30}$$

where $R_\pm = R_{\gamma_3} \pm iR_{\gamma_1}$ $f_\pm = (B \pm \sqrt{B^2 + 8g^2})/g$ and $d_\pm = 8 + f_\pm^2$. The energies ϵ_1 and ϵ_2 correspond to the eigenstates obtained by mixing the positive angular momentum magnons and phonons, while the energies ϵ_3 and ϵ_4 correspond to the eigenstates that mix negative angular momentum magnons and phonons. These states are respectively shown in Figs. S12a and S12b. Depending on the sign of g either the lower states ϵ_1 and ϵ_3 in each magnon polaron pair, or the upper states ϵ_2 and ϵ_4, show a cross-over in polarization dependence as a function of B.

The polarization dependence of state $|\epsilon_1\rangle$ is determined by the sign of the difference $|2R_+ - if_+R_m| - |2R_+|$. This sign in turn is determined by the imaginary part of $2R_+ - if_+R_m$, since the real parts of the two terms are equal. A similar argument holds for states ϵ_2 to ϵ_4. Since f_+ is a monotonic function, $|2R_+ - if_+R_m|$ might cross the line $|2R_+|$ at most once. If such a crossing happens the state changes its polarization dependence as a function of B, while if the crossing doesn't happen the polarization dependence is constant.

To check if $|2R_+ - if_\pm R_m|$ crosses the line $|2R_+|$, we work out the asymptotic values for $B \to \pm\infty$. Expanding the square root in $f_\pm$ we find $\sqrt{B^2 + 8g^2} = |B| + 4g^2/|B| + \mathcal{O}(B^{-2})$,

and thus

$$\mathrm{Im}(2R_+ - if_\pm R_m) = 2R_{\gamma_1} - \frac{B \pm (|B| + 4g^2/|B|)R_m}{g}. \tag{S31}$$

Taking the positive sign, we find the above expression goes to $2BR_m/g$ as $B \to \infty$, and to $4gR_m/|B| - 2R_{\gamma_1}$ for $B \to -\infty$. The former is always larger in magnitude than $2R_{\gamma_1}$, while the latter is smaller (larger) in magnitude than $2R_{\gamma_1}$ for $g > 0$ ($g < 0$). A similar analysis for the negative sign shows the above expression tends to $2|B|R_m/g$ as $B \to -\infty$, and to $-4gR_m/B - 2R_{\gamma_1}$ for $B \to \infty$. Again the former is always larger in magnitude than $2R_{\gamma_1}$, while the latter is larger (smaller) in magnitude than $2R_{\gamma_1}$ for $g > 0$ ($g < 0$). Thus, depending on the sign of g, the polarization dependence of either state ϵ_1 or ϵ_2 changes as a function of B. The same conclusion holds for ϵ_3 and ϵ_4 as well.

We can further work out the crossing point, if it exist, by solving the equation $|2R_+ - if_+R_m| - |2R_+| = 0$. This gives

$$B = \pm g\left(\frac{2R_{\gamma_1}}{R_m} - \frac{R_m}{R_{\gamma_1}}\right). \tag{S32}$$

We note that for $R_m/R_{\gamma_1} \ll 1$ the crossing point is for large positive B and the spectrum looks as in Fig. 5d. However, for $R_{\gamma_1}/R_m \ll 1$ the crossing point is for large negative B and the spectrum looks like that of the uncoupled system.

S10. Point group symmetries of paramagnetic and zigzag states in monolayer FePSe$_3$. The point group symmetry of paramagnetic FePSe$_3$ is $D_{3\mathrm{d}}$, while the symmetry group of the zigzag state is $C_{2\mathrm{h}}$.[4,5] We note that the point group of the paramagnetic state has a Raman active two-dimensional representation E_g that decomposes into the direct sum $E_\mathrm{g} = A_\mathrm{g} \oplus B_\mathrm{g}$ when breaking the symmetry to the subgroup $C_{2\mathrm{h}}$. Thus, any circular phonons of the paramagnetic state would appear as the sum of an A_g and a B_g mode.

REFERENCES

[1] A. R. Wildes, M. E. Zhitomirsky, T. Ziman, D. Lançon, and H. C. Walker, Evidence for biquadratic exchange in the quasi-two-dimensional antiferromagnet FePS3, Journal of Applied Physics **127**, 223903 (2020).

[2] G. D. Mahan, *Many-Particle Physics* (Springer US, 2000).

[3] L. Zhang and Q. Niu, Angular momentum of phonons and the einstein–de haas effect, Phys. Rev. Lett. **112**, 085503 (2014).

[4] J.-U. Lee, S. Lee, J. H. Ryoo, S. Kang, T. Y. Kim, P. Kim, C.-H. Park, J.-G. Park, and H. Cheong, Ising-type magnetic ordering in atomically thin FePS3, Nano Letters **16**, 7433 (2016).

[5] A. Hashemi, H.-P. Komsa, M. Puska, and A. V. Krasheninnikov, Vibrational properties of metal phosphorus trichalcogenides from first-principles calculations, The Journal of Physical Chemistry C **121**, 27207 (2017).
